# The Risk of Orofacial Cleft Lip/Palate Due to Maternal Ambient Air Pollution Exposure: A Call for Further Research in South Africa

**DOI:** 10.5334/aogh.4007

**Published:** 2023-01-27

**Authors:** Caradee Y. Wright, Thandi Kapwata, Bianca Wernecke, Helen Malherbe, Kurt-W Bütow, Natasha Naidoo, Rebecca M. Garland, Anzel de Lange, Gareth E. Murray, OPERATION SMILE

**Affiliations:** 1 Environment and Health Research Unit, South African Medical Research Council, Pretoria, ZA; 2 Department of Geography, Geoinformatics and Meteorology, University of Pretoria, Pretoria, ZA; 3 Environment and Health Research Unit, South African Medical Research Council, Johannesburg, ZA; 4 Department of Environmental Health, Faculty of Health Sciences, University of Johannesburg, Johannesburg, ZA; 5 Rare Diseases South Africa, The Station Bryanston, 63 Peter Place, Sandton, ZA; 6 Department of Maxillo-Facial and Oral Surgery, University of Pretoria, and Life-Wilgers Hospital, Pretoria, ZA; 7 Department of Geography and Environmental Studies, University of Limpopo, Limpopo, ZA; 8 OPERATION SMILE, Building 17, 103–104 Waverley Business Park, 5 Wyecraft Rd. Mowbray, Cape Town, ZA

**Keywords:** air pollution, congenital disorder, birth defect, orofacial cleft lip/palate, craniofacial anomalies, environmental health, particulate matter

## Abstract

**Background:**

Despite being underreported, orofacial cleft lip/palate (CLP) remains in the top five of South Africa’s most common congenital disorders. Maternal air pollution exposure has been associated with CLP in neonates. South Africa has high air pollution levels due to domestic burning practices, coal-fired power plants, mining, industry, and traffic pollution, among other sources. We investigated air pollutant levels in geographic locations of CLP cases.

**Methods:**

In a retrospective case series study (2006–2020) from a combined dataset by a Gauteng surgeon and South African Operation Smile, the maternal address at pregnancy was obtained for 2,515 CLP cases. Data from the South African Air Quality Information System was used to calculate annual averages of particulate matter (PM) concentrations of particles < 10 µm (PM_10_) and < 2.5 µm (PM_2.5_). Correlation analysis determined the relationship between average PM_2.5_/PM_10_ concentrations and CLP birth prevalence. Hotspot analysis was done using the Average Nearest Neighbor tool in ArcGIS.

**Results:**

Correlation analysis showed an increasing trend of CLP birth prevalence to PM_10_ (CC = 0.61, 95% CI = 0.38–0.77, p < 0.001) and PM_2.5_ (CC = 0.63, 95% CI = 0.42–0.77, p < 0.001). Hot spot analysis revealed that areas with higher concentrations of PM_10_ and PM_2.5_ had a higher proclivity for maternal residence (z-score = –68.2, p < 0.001). CLP birth prevalence hotspot clusters were identified in district municipalities in the provinces of Gauteng, Limpopo, North-West, Mpumalanga, and Free State. KwaZulu-Natal and Eastern Cape had lower PM_10_ and PM_2.5_ concentrations and were cold spot clusters.

**Conclusions:**

Maternal exposure to air pollution is known to impact the fetal environment and increase CLP risk. We discovered enough evidence of an effect to warrant further investigation. We advocate for a concerted effort by the government, physicians, researchers, non-government organizations working with CLP patients, and others to collect quality data on all maternal information and pollutant levels in all provinces of South Africa. Collaboration and data sharing for additional research will help us better understand the impact of air pollution on CLP in South Africa.

## 1. Introduction

The global birth prevalence estimates for orofacial cleft lip and palate (CLP) stand at 0.6–2.6 per 1,000 live births [1]. A supposedly more accurate global birth prevalence of 0.45 per 1,000 live births (95% Confidence interval (CI): 0.38–0.52) was calculated from a recent meta-data analysis [2]. Africa has reported a lower CLP birth prevalence than the rest of the world [3]. South African CLP birth prevalence is estimated at 0.3 per 1,000 live births [3]. Birth prevalence estimates in low- to middle-income countries (LMICs) are less accurate than in high-income countries (HICs), where more active CLP surveillance systems and research programs exist [3,4]. A higher reported birth prevalence of CLP in African descendants outside of Africa, as well as several genetic studies, suggests an underreporting of CLP in Africa [5,6,7,8].

CLP patients have a higher mortality risk and live with disabilities such as speech impediments, physical inadequacies in their appearance, and psychosocial issues [9,10]. Patients require a multidisciplinary approach to care from birth and lifelong management [10]. Among the difficulties faced by children with CLP are nutritional difficulties due to problems ingesting food, affecting their overall health [11]. Death from malnutrition with CLP as a root cause is underreported because the cause of death on death certificates is often listed as malnutrition [12]. Given the aforementioned considerations, preventing CLP where possible would prevent unnecessary suffering and financial strain of those affected.

CLP malformation complications can cause serious problems with negative social and economic consequences [13]. Financial constraints are common among CLP cases in LMICs, causing delays in surgical interventions and, in some cases, no facial repair [14,15]. A study involving 36,384 patients and 389 African surgeons from 33 African countries reported that patients at both ends of the CLP severity spectrum do not seek CLP repair [15]. The surgeons documented fewer females with isolated cleft palates, and fewer than expected severely affected patients with associated anomalies [15]. Despite the fact that gross morbidity and mortality can be avoided with early CLP detection and treatment [13], high rates of unrepaired CLP cases are still reported in LMICs [12].

It was previously assumed that air pollutants are diluted in public places and would expose a developing fetus to lower concentrations, placing them at risk solely during the brief period when the palate and lips develop [16]. However, the global concentration of particulate matter (PM) < 10 µm (PM_10_) and < 2.5 µm (PM_2.5_), and other air pollutants are increasing annually [17]. Concurrently, the annual mortality due to ambient air pollution is increasing, with a 36% increase from 1990 to 2013 [18]. Thus, it has become more important to consider the concentrations of air pollutants. Maternal air pollution exposure has been identified as a risk factor for fetal mortality and congenital disorders [16]. Congenital disorders such as CLP have a well-reported heritability rate [19], but it is also recognized that air pollutants may impact the fetal environment and adversely affect embryonic development [20]. In some population-based studies, it has been difficult to validate air pollutants as major teratogens causing CLP [21,22]. There are many confounding factors, such as exposure to high temperature [23,24], maternal smoking [25], and maternal malnutrition [26,27], among others [22]. Disparities in the literature result from environmental, methodological, study population and genetic variations [22], as well as differences in air pollutant concentrations depending on location. Additionally, CLP is often studied alongside a wide range of congenital disorders. Nonetheless, important evidence on the environmental factors that alter gene expression, triggering CLP, have been published [28].

Significant progress has been made in understanding the pathogenic mechanism that leads to CLP, revealing the relationship between genetic susceptibility and air pollutants in particular [22]. High PM_10_ exposure and two CYP gene variants were linked to an increased risk of CLP [22]. Studies from across the globe have found associations between several air pollutants and CLP risk. Several studies in the United States have investigated the risk of CLP in the presence of either elevated concentrations of PM_10_, PM_2.5_, or both [29,30,31,32]. Mothers who visited referral hospitals near areas with higher pollutant concentrations during their first eight weeks of pregnancy had a higher risk of having babies affected by CLP [32]. CLP was associated with PM_10_ (Odds Ratio (OR) 1.72, 95% CI: 1.12–2.66), PM_2.5_ (OR 1.74, 95% CI: 1.15–2.64), SO_2_ (OR 1.93, 95% CI: 1.16–3.21), NOx (OR 3.64, 95% CI: 1.73–7.66), and CO (OR 2.24, 95% CI: 1.21–4.16) [32]. In Texas, Poisson regression showed a significant relationship between lower environmental quality where mothers lived and higher CLP birth prevalence (PR 1.54, 95% CI: 1.04–2.28) [33]. Thus, with the increasing concentrations of air pollutants in HICs, more recent evidence has been published on the link between air pollutants and CLP risk.

There have been fewer studies on the links between CLP and environmental air pollutants in LMICs than in HICs. Studies on maternal tobacco smoking linked to increased odds of bearing an infant with CLP have taken precedence [34]. While the majority of research on environmental determinants of CLP in LMICs has been conducted in China, PM_10_ and PM_2.5_ have not been the primary focus of many studies [34,35,36,37,38,39]. Nonetheless, the LMIC studies that exist offer evidence that air pollutants are significant risk factors for CLP. A Mongolian study tracked the exposure of pregnant women to environmental pollutants during their first eight weeks of pregnancy [40]. This study showed significant associations between CLP and PM_2.5_ (OR 2.25, 95% CI: 0.62–8.1), SO_2_ (OR 2.6, 95% CI: 0.61–11.12), and CO (OR 2.83, 95% CI: 0.92–8.72) [40]. Interestingly, a spatial statistical scan in Mexico revealed that higher PM_10_ concentrations were associated with increased CLP risk [41]. Spatial clusters comprised up to 12 CLP cases in a 1.2km radius, with 95% of CLP cases found statistically closer to higher PM_10_ exposure [41]. More studies comprising spatial scanning could substantively verify causal links between PM and CLP risk.

There are no substantial African studies demonstrating the association between air pollutants and CLP risk. Related African studies have focused on socioeconomic status, educational level, cigarette smoking, alcohol consumption, indoor cooking with charcoal, and the use of multivitamins, among other factors [42,43,44]. Data collection on air quality in several Sub-Saharan African countries is reportedly insufficient to inform science-driven health research [45].

Considering the growing health concerns from ever-increasing air pollutant concentrations [46], this study sought to explore maternal ambient air pollutant exposure as a major risk factor for CLP. Specifically, we studied the association between PM_10_ and PM_2.5_ levels and CLP birth prevalence in the districts of mothers of infants with CLP. The air quality in the major provinces of South Africa, namely Gauteng, Mpumalanga, Western Cape, and KwaZulu-Natal was matched to the place of residence of mothers with children affected by CLP.

## 2. Methods

### Air pollution data

Particulate matter (PM) data with an aerodynamic diameter of 2.5 (PM_2.5_) and 10 (PM_10_) micrometers between 2006 and 2020 were sourced from the South African Air Quality Information System (https://saaqis.environment.gov.za/) through scripted POST (a method used to send data to a destination using the Internet) requests. Data available in hourly averages per day were downloaded, filtered, and merged into comprehensive and continuous datasets for the entire study period for each ambient air quality monitoring station for the two listed pollutants. The data sets were quality controlled in the web-based user interface Jupyter Lab, considering negative values, missing data, and outliers. Annual averages were calculated using the 99^th^ and 98^th^ percentile for PM_10_ and PM_2.5_, respectively, and only if data availability for a monitoring station exceeded 50%. This was done to match the temporal resolution of the health data to enable a direct correlation between PM concentrations and CLP birth prevalence. Though 50% data availability is generally considered low, the threshold for inclusion of an air quality monitoring station’s data in this study was lowered to ensure a larger geographical representation of ambient air quality. To provide an overview of annual PM concentrations over the study period at a provincial level, descriptive statistics, including mean, standard deviation, and median and interquartile range (IQR) were conducted. The 50% data availability threshold as well as the provincial concentration averages are considered limitations of the study, as uncertainties are introduced when data used may not be considered representative due to lacking data or due to high spatial variability.

### Study population

A retrospective cohort of patients with CLP for the period 2006–2020 was obtained from two databases and combined into one dataset. The first database consisted of patient records of 4,804 patients treated at a hospital in Pretoria, Gauteng by a maxillo-facial and oral surgeon. The maternal place of residence during the pregnancy was extracted from the surgeon’s database of patients (the database is self-managed by the surgeon and comprises all the patients he treats). All patients were included regardless of age.

The second database was provided by Operation Smile South Africa and comprised 485 individuals. Operation Smile is an international medical charity that raises funds to provide free surgical procedures for children and young adults born with CLP. Cases are screened to confirm the diagnosis by medical practitioners including pediatricians, nurses, anesthesiologists, and surgeons all formally licensed, trained and certified to work with patients at the mission site.

For all cases in both databases, CLP was classified into eight categories: cleft lip (CL); cleft lip and cleft alveolus (CLA); cleft lip, cleft alveolus, hard palate cleft and soft palate cleft (CLAP); hard palate cleft (hP); hard palate cleft and soft palate cleft (hpsP); soft palate cleft (sP); combination cleft (CL or CLA and sP without hP); and oblique (involves soft tissue and/or skeleton around the eye). Patients were included in our database if they were accompanied by their biological mother (18 years or older) and the mother reported their place of residence (not necessarily their place of residence during pregnancy, and this is discussed in the limitations).

A total of 5,289 cases of CLP were merged from the two datasets; however, only 2,515 could be geocoded due to missing information for maternal place of residence during pregnancy. Half the CLP cases were in Gauteng province (52%) since the larger of the two databases used was from a surgeon located in Gauteng (although 39% of his patients were from other provinces).

Research ethics approval for the study was granted by the University of Pretoria Research Ethics Committee (NAS 142/2020 and NAS 334/2020).

### Data analysis

Data was first managed in Microsoft Office™ packages: Microsoft Excel™ and Microsoft Access™. Cases of CLP were assigned geographic coordinates in ArcGIS 10.3. Cases from maternal place of residence were then aggregated to the district municipality level. Life-time birth prevalence of CLP per district municipality was then calculated per 1 000 live births. The following equation incorporating yearly live births from Statistics South Africa for the period 2006 to 2020 (Stats SA 2020) was used as the denominator:

Birth\ Prevalence = \frac{{Number\ of\ CLP\ cases}}{{Total\ live\ births\ in\ district\ municipality}}\,\, \times \,\,1,000

### Statistical Analysis

#### Correlation Analysis

Correlation analysis, conducted using STATA version 15 [47], was used to determine the link between annual average PM_2.5_ and PM_10_ concentrations at a site and CLP birth prevalence at the district municipality level. The PM_2.5_ and PM_10_ concentrations obtained from air quality monitoring stations that were included in the analysis had more than 50% data availability. Correlation coefficients (CC) are reported with the associated 95% confidence intervals (CI) and p-values (α < 0.05) denoting whether data values are statistically significant.

#### Hot spot analysis

The Average Nearest Neighbor tool in ArcGIS was used to measure the distribution of CLP cases to determine whether cases were clustered or uniformly spaced and to identify possible patterns in clusters. The Average Nearest Neighbor tool measures the distance between the centroid of each feature and its nearest neighbor’s centroid. It then averages all these nearest-neighbor distances to calculate a ratio using the observed average distance divided by the expected average distance. If the ratio is less than 1, the pattern exhibits clustering. If it is greater than 1, the trend is toward dispersion.

The Hot Spot Analysis tool in ArcGIS 10.3 was used to identify statistically significant spatial clusters of high values (hot spots) and low values (cold spots) of CLP birth prevalence. The results provide z-scores and p-values. Z-scores are standard deviations and very high or very low (negative) z-scores are associated with very small p-values and are found in the tails of the normal distribution. For statistically significant positive z-scores, the larger the z-score is, the more intense the clustering of high values (hot spot). For statistically significant negative z-scores, the smaller the z-score is, the more intense the clustering of low values (cold spot). Confidence levels were derived from z-scores of hot and cold spots and were based on 90%, 95%, and 99%.

## 3. Results

### CLP descriptives

Since CLP surgery is a specialized area of medicine, South African CLP patients are referred to specified centers equipped to handle their cases. Thus, accurate databases were accessible for data acquisition. In our study, the main center for CLP surgery was based in Gauteng Province and patients from other provinces were also treated at the facility. Although a smaller subset of data, South African Operation Smile included some CLP cases from the population that were not able to afford corrective surgery.

### Exposure analysis for 2006 to 2020

Only annual datasets from air quality monitoring stations with data availability > 50% were included for analysis, amounting to data from 74 air quality monitoring stations for PM_2.5_ and 98 air quality monitoring stations for PM_10_. The South African government has identified poor air quality in parts of Gauteng and Mpumalanga provinces, as well as the Vaal Triangle, which includes parts of Gauteng and Northern Free State [48,49]. When analyzed by province, Gauteng and Mpumalanga Provinces showed the highest number of ambient air quality monitoring stations by absolute number, substantiated by the inclusion of these areas in the country’s air quality management priority areas (i.e., Highveld Priority Area, Waterberg-Bojanala Priority Area, and the Vaal Triangle Airshed Priority Area) and the increased need to monitor air quality. On average, when considering annual average PM_2.5_ and PM_10_ concentrations per province across the country, Gauteng, Free State, Mpumalanga, and North-West were found to have the highest average annual PM_2.5_ and PM_10_ concentrations, respectively (Table 1). There were years when PM data was not collected at specific sites (Refer to Supplementary Material).

**Table 1:** Overview of PM_2.5_ and PM_10_ annual average data at South African Ambient Air Quality Monitoring Stations at 50% data availability (South African National Ambient Air Quality Standards (NAAQS) current limit values for annual averages: PM_2.5_ = 20 µg/m^3^; PM_10_ = 40 µg/m^3^).


	PM_2.5_			PM_10_		
	
PROVINCE	STATIONS PER PROVINCE*	MEAN ± STD DEV	MEDIAN ± IQR	STATIONS PER PROVINCE*	MEAN ± STD DEV	MEDIAN ± IQR

Eastern Cape	4	9 (4)	11 (2)	5	21 (9)	23 (8)

Free State	5	**34 (8)**	**32 (8)**	5	**70 (25)**	**59 (36)**

Gauteng	15	**35 (18)**	**32 (11)**	25	**59 (25)**	**55 (23)**

KwaZulu-Natal	11	19 (13)	17 (5)	14	32 (13)	28 (17)

Limpopo	8	18 (13)	14 (7)	8	39 (19)	32 (24)

Mpumalanga	22	**23 (8)**	**22 (8)**	26	**51 (21)**	**50 (26)**

North-West	4	**25 (20)**	14 (12)	4	**44 (18)**	**42 (36)**

Northern Cape	1	4 (–)	– (–)	1	7 (–)	–(–)

Western Cape	4	10 (5)	8 (4)	10	30 (9)	31 (11)


* Number of stations which met the > 50% data availability threshold at least once over the period. Not all stations had data every year.

When zooming in on Mpumalanga and Gauteng, exceedances of the current annual PM_2.5_ and PM_10_ South African National Ambient Air Quality Standards (NAAQS) were evident in both provinces between 2007 and 2020 (Figures S1 and S2 in Supplementary Material). Current annual NAAQS were used to enable direct comparison between sites over the study period.

### Correlation Analysis

**Figure 1:**
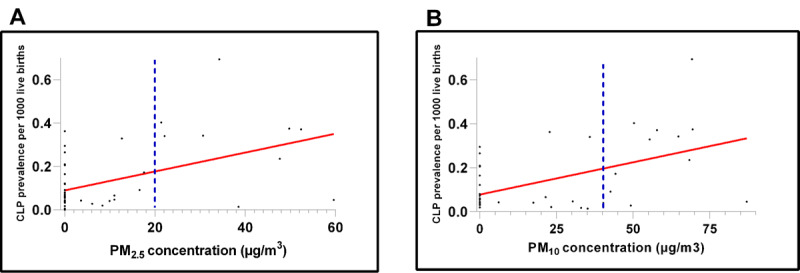
Scatter plots of average annual **(A)** PM_2.5_ and **(B)** PM_10_ and CLP birth prevalence per 1,000 live births from 2006 to 2020. The dashed blue vertical lines represent the current South African NAAQS of 20 and 40 µg/m^3^ for annual PM_2.5_ and PM_10_, respectively.

The scatter plots in Figure 1 show linear positive trends between average yearly PM_2.5_ and CLP birth prevalence per 1,000 live births. The same was observed for PM_10_. A clustering of CLP affected births (higher birth prevalence) was evident at PM_2.5_ concentrations below 30 µg/m^3^; therefore, the correlation analysis was limited to that subset of data points.

Results showed statistically significant moderate positive correlations between PM_2.5_, PM_10_ and CLP birth prevalence (correlation coefficient (CC) = 061, 95% CI = 0.38–0.77, p = < 0.001 and CC = 0.63, 95% CI = 0.42–0.77, p = < 0.001, respectively), when PM concentrations were ≤ 30 µg/m^3^.

### Spatial Scanning using Hot Spot Analysis

The results of the Average Nearest Neighbor Analysis showed that the nearest neighbor ratio was less than 1, so this pattern exhibited statistically significant clustering of CLP cases (p < 0.001). The z-score of –68.2 shows that there is a less than 1% chance that the clustering patterns observed in the data are due to random chance (Figure S2 in Supplementary Material).

**Figure 2:**
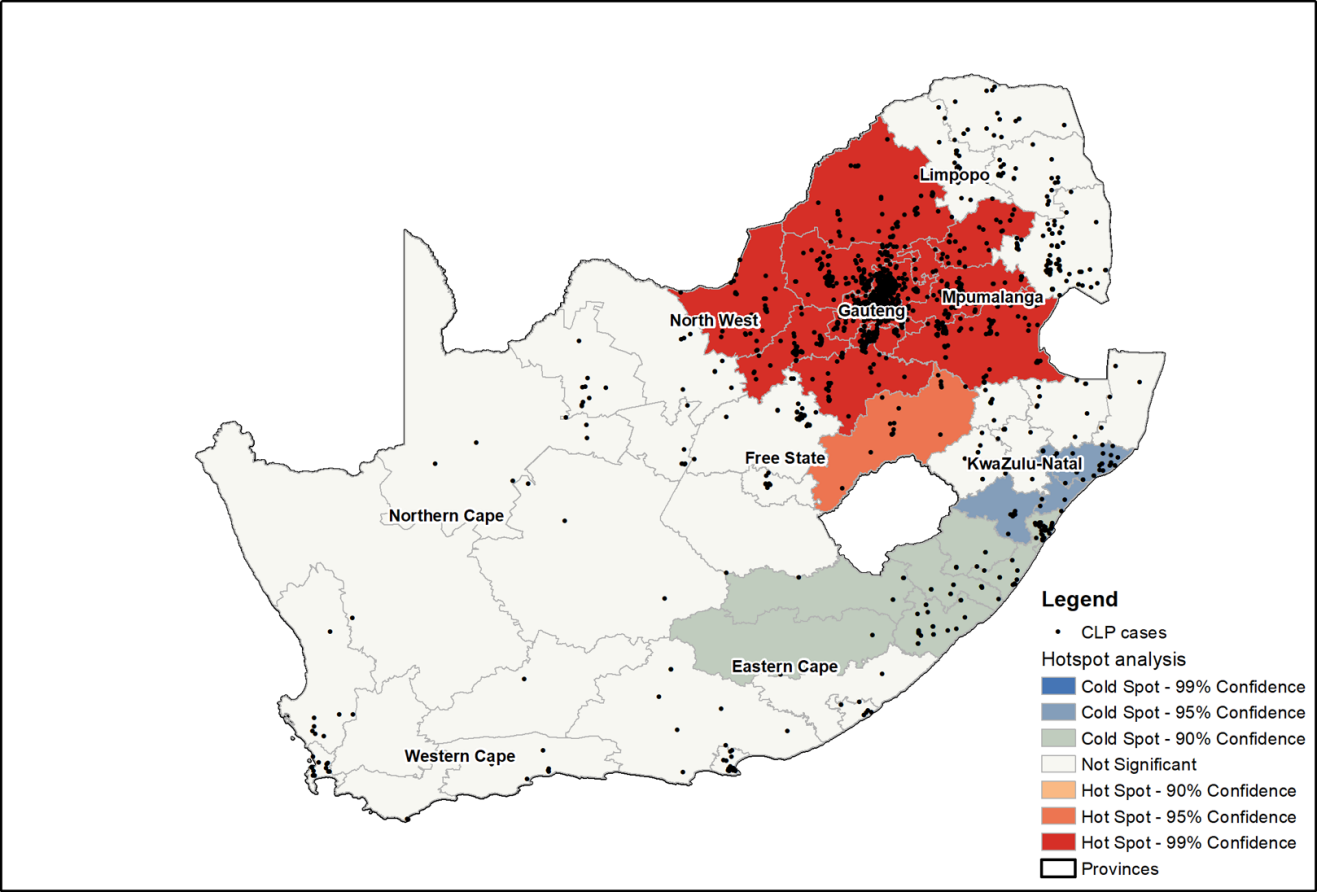
Location of all CLP cases in our dataset overlaid by the findings of hot and cold spots of CLP birth prevalence per 1,000 live births by district municipality. The table for CLP birth prevalence for each district municipality per 1,000 live births is given in the Supplementary Material.

Significant hot spot clusters for CLP birth prevalence were identified in the inland provinces of Gauteng and parts of Limpopo, North-West, Mpumalanga and Free State provinces (Figure 2). Significant cold spot clusters were located along the coastal provinces of KwaZulu-Natal and Eastern Cape. Other parts of the country where data was available did not have any significant clusters of CLP birth prevalence. It is important to note that one of the statistically significant hot spots, the Gert Sibande district in Mpumalanga province, had the second highest CLP birth prevalence rate documented (0.40 per 1,000 live births), although it only had the seventh highest number of CLP cases.

## 4. Discussion

In LMICs, few studies focus on the component of the CLP burden of disease caused by maternal exposure to ambient air pollutants [40,41]. However, limited studies in both LMICs and HICs demonstrate that areas with higher levels of PM_10_, PM_2.5_, and other air pollutants have a higher birth prevalence of CLP. This study presents the first and largest South African dataset to provide preliminary evidence correlating higher ambient air pollutant levels to greater birth prevalence of CLP. According to the findings of the present study, as PM_10_ and PM_2.5_ concentrations rise, so does the extent of CLP birth prevalence. The birth prevalence of CLP increased with rising concentrations of PM_10_ (CC = 0.61) and PM_2.5_ (CC = 0.63). At the provincial and district levels, the location of the mothers’ residence noted in the database was positively related with higher PM_10_ and PM_2.5_ concentrations.

The mean PM_10_ and PM_2.5_ concentrations in all nine of South Africa’s provinces were examined. The highest PM_10_ and PM_2.5_ mean concentrations were found in the Free State and Gauteng provinces, followed closely by Mpumalanga, North-West, and Western Cape. PM_10_ and PM_2.5_ concentrations at individual sites exceeded NAAQS in the Free State, Gauteng, Mpumalanga, and the North-West. This is in accordance with a recent South African study on PM_10_ levels exceeding the World Health Organization (WHO) PM_10_ air quality standards. Between 2010 and 2017, Gauteng had daily PM_10_ concentrations that were 5.7 times higher than the WHO standard of 15 g/m^3^ [45]. Thus, there is an agreement with the present study’s data that estimated a mean PM_10_ concentration of 59 ± 25 µg/m^3^ in Gauteng (Table 1). The South African NAAQS PM_10_ standard stands at 40ug/m^3^ and has been exceeded in certain areas, including parts of Gauteng, Mpumalanga, and parts of the North-West [50]. Likewise, the NAAQS PM_2.5_ standard of 25μgm^–3^ has been exceeded in these areas [51]. The most significant contributing emissions include increased vehicle emissions, mine trailing, domestic combustion, electricity generation, and regional industry. Interestingly, the inland areas of South Africa have higher PM_10_ and PM_2.5_ concentrations than coastal regions [45].

The present study revealed that there was a higher chance of mothers with CLP-affected infants in provinces with higher levels of air pollutants. There was a tendency for CLP cases to cluster in certain geographic locations as opposed to a randomly dispersed pattern (z-score = – 68.2, p < 0.001) (Refer to Figure S1). Hotspot analysis confirmed that higher concentrations of PM_10_ and PM_2.5_ were associated with specified geographic locations of mothers with CLP-affected infants. As a result, “hotspot clusters” of cases of CLP were identified in Gauteng, Limpopo, North-West, Mpumalanga, and Free State. Areas with fewer cases of CLP, such as KwaZulu-Natal and the Eastern Cape, had lower PM_10_ and PM_2.5_ concentrations and were termed “cold spot clusters.” Air pollutant concentrations in inland and coastal geographical locations are affected by wind speed, precipitation, relative humidity, population density and industrial activities [52]. Air pollution levels at Durban’s port in KwaZulu-Natal have a distinct seasonal pattern, but also depend on port activities, motor vehicle traffic, and industrial activities [53].

The results of this investigation can be compared to previous information on cleft etiology and cross-referenced with results from previous studies using different methods. It has been biologically proven that PM_2.5_ and other pollutants cross the placenta and exhibit genetic toxicity [54]. Thus, it is plausible that this and other studies have found an association between maternal exposure to air pollutants during pregnancy and CLP. Although CLP risk was not directly analyzed in the present study, our data echoes more sophisticated studies using spatial statistical analyses that link CLP birth prevalence to areas where there are higher concentrations of air pollutants [41]. Sophisticated methods of spatial scanning were demonstrated as highly effective for defining high CLP birth prevalence hotspots [55]. A large multicenter study in the United States found a significant association between maternal exposure to PM_2.5_ during early gestation and an etiology of cleft lip alone (OR 1.43 95% CI: 1.11–1.86) [31]. The analysis was adjusted for several confounding factors including maternal education, smoking status during pregnancy, and others [31]. Every 10 µg/m^3^ increase in county-level PM_2.5_ concentration was estimated to increase the risk of having a baby with CLP by 43%, with greater probability of CLP during gestational weeks 5 to 10 [31]. More South African studies, similar to those conducted in the aforementioned HICs and LMICs, are required to determine CLP risk in response to air pollution.

### Strengths

National data for both outcome and exposure were systematically collected throughout the study period and is a key strength of the study. The ecological study design allows researchers to investigate the contextual effect of an environmental risk factor on a health outcome in a complex, real-world scenario, while avoiding selection bias associated with groups of cases in individual-level/case studies. The data presented in this study is sourced from PM_10_ and PM_2.5_ concentrations and cases of CLP that span a fifteen-year period (2006–2020). Thus, the limitation of missing PM_10_ and PM_2.5_ exposure data and maternal place of residence during pregnancy was mitigated since more data points were available. The optimized data availability enhanced statistical significance of the analyses performed.

Pollution levels vary greatly spatially, and where CLP cases did not live close to a monitoring station adds a degree of uncertainty. The legitimacy of the dataset relied both on an extended study period and on a high number of air pollution monitoring stations used, including 74 air quality monitoring stations for PM_2.5_ and 98 for PM_10_. Air pollution measurements included the average distance of study participants from these stations, the number and density of monitoring stations, and the frequency of measurements recorded at sites.

### Limitations

The present study was a case series using data from one surgeon and one organization (other data exist; however, we did not succeed in attaining them for this study, but will do for future studies), and participants were not randomly selected; they were included in our dataset if they had CLP and the mother’s residence was known. Birth prevalence may have been underestimated since the data were only from two sources and are not fully representative of the true country-wide CLP birth prevalence. Nonetheless, the datasets represented the majority of CLP cases that would be referred to the surgeon in Gauteng and to Operation Smile.

The low final number of cases of CLP recruited is indicative of the lack of available data for epidemiological and scientific analyses. Additionally, the understanding of a time-series trend in exposure data across sites was compromised. This contrasts with HICs. A retrospective cohort study of 124,842 births in a 14-year period in rural Colorado showed maternal proclivity to natural gas wells where there were higher rates of infants with congenital disorders [56]. More PM_10_ and PM_2.5_ data of adequate quality, as well as spatial coverage of CLP case characteristics, are desperately needed.

The 50% threshold for air quality data availability adds further uncertainty as it may not be representative of a full year (e.g., if winter data are missing, pollution peaks may not be included). The provincial averages reported in this study represent an average of the air quality monitoring stations used in the respective province.

The reported maternal residence during pregnancy could not be verified. Since this study was exploratory, the demographics of our sample participants were not investigated, including maternal age, ethnicity, teratogenic exposure (smoking, alcohol, prescribed and recreational drugs), diet, socioeconomic status, infant gender, gestational age, and season of conception. Household or indoor air pollution should be considered in future studies as there is evidence of differences in indoor and outdoor PM levels [57]. Despite the limitations, our results provide some insight into the effects of PM_2.5_ and PM_10_ concentrations on CLP birth prevalence in South African provinces during the study period.

### Future research

This study demonstrates the importance of linking poor air quality in specific geographic areas with CLP risk. Thus, further studies using more intensive spatial clustering techniques should identify the true impact of increased PM_10_ and PM_2.5_ and associated genetic factors on CLP risk. There are a few interesting factors to study, including the influence of seasonality on air pollution and the effect on CLP birth prevalence and maternal exposure to indoor pollutant levels throughout pregnancy in high-risk locations in South Africa. In addition, seasonality of exposure with time trends can be explored. This study has notable strengths and several limitations, and since it is a preliminary study and a call for more research, the abovementioned limitations should serve as the foundation for better study designs.

## Conclusion

Our findings add to the growing body of research on the links between periconceptional air pollution exposure and CLP risk. Maternal exposure to air pollution is known to threaten fetal growth [54]. However, little has been done to investigate how such exposure may be affecting the South African population. We found sufficient evidence of an effect worthy of further investigation. We call for a concerted effort between government, physicians, researchers, and non-governmental organizations, like Operation Smile and others, for thorough data collection of all maternal information, collaboration and data sharing and additional research to understand the impact of air pollution more fully on CLP in South Africa. Unlike other risk factors such as smoking and cooking, environmental air pollution is not easily modifiable. Thus, it has become critical to address this factor as a significant threat to human health in South Africa.

## Additional File

The additional file for this article can be found as follows:

10.5334/aogh.4007.s1 Supplementary material. Table S1 and Figures S1 to S3.
