# Supplementary material for: The Risk of Orofacial Cleft Lip/Palate Due to Maternal Ambient Air Pollution Exposure: A Call for Further Research in South Africa
Source: Ann Glob Health. 2023 Jan 27;89(1):6. doi: 10.5334/aogh.4007 (PMC9881443; doi:10.5334/aogh.4007)
Supplement: Supplementary material. — Table S1 and Figures S1 to S3. [file agh-89-1-4007-s1.pdf]

Supplementary material

Table S1. CLP birth prevalence per 1,000 live births for the 52 districts by province in South Africa.

**Province**	**District municipality**	**Total live births (2006–2020)**	**Total CLP cases (2006–2020)**	**Birth Prevalence**
Eastern Cape	Alfred Nzo	235,367	19	0.080
	Amathole	202,611	1	0.004
	Buffalo City	254,308	7	0.027
	Sarah Baartman (Cacadu)	98,641	9	0.091
	Chris Hani	222,159	10	0.045
	Joe Gqabi	81,537	5	0.061
	Nelson Mandela Bay	297,058	14	0.047
	O.R. Tambo	512,399	37	0.072
Free State	Fezile Dabi	125,543	43	0.342
	Lejweleputswa	165,311	34	0.205
	Mangaung	250,577	8	0.031
	Thabo Mofutsanyane	220,448	16	0.072
	Xhariep	25,266	1	0.039
Gauteng	City of Johannesburg	985,426	232	0.235
	City of Tshwane	930,494	646	0.694

	Ekurhuleni	880,849	260	0.295
	Sedibeng	247,990	93	0.375
	West Rand	209,432	76	0.362
KwaZulu-Natal	Amajuba	153,691	10	0.065
	eThekwini	997,087	45	0.045
	iLembe	153,201	3	0.019
	King Cetshwayo	333,631	55	0.164
	Harry Gwala (Sisonke)	169,276	6	0.035
	Ugu	227,498	9	0.039
	Umgungundlovu	256,733	9	0.035
	Umkhanyakude	239,689	5	0.020
	Umzinyathi	190,115	7	0.036
	Uthukela	212,005	8	0.037
	Zululand	301,214	9	0.029
Limpopo	Capricorn	447,763	53	0.118
	Mopani	393,998	26	0.065
	Sekhukhune	337,385	27	0.080
	Vhembe	467,028	19	0.040
	Waterberg	245,207	65	0.265

Mpumalanga	Ehlanzeni	648,269	136	0.209
	Gert Sibande	258,112	104	0.402
	Nkangala	309,825	115	0.371
North-West	Bojanala	371,182	122	0.328
	Dr Kenneth Kaunda	205,863	70	0.340
	Dr Ruth Segomotsi Mompati	158,529	9	0.056
	Ngaka Modiri Molema	191,203	33	0.172
Northern Cape	Frances Baard	136,421	6	0.043
	John Taolo Gaetsewe	82,232	10	0.121
	Namakwa	23,623	1	0.042
	Pixley ka Seme	55,565	3	0.053
	Z F Mgcawu	73,380	6	0.081
Western Cape	Cape Winelands	205,462	0	0.000
	Central Karoo	18,264	1	0.054
	City of Cape Town	1037,920	14	0.013
	Eden	140,963	6	0.042
	Overberg	56,799	1	0.017
	West Coast	87,474	8	0.091

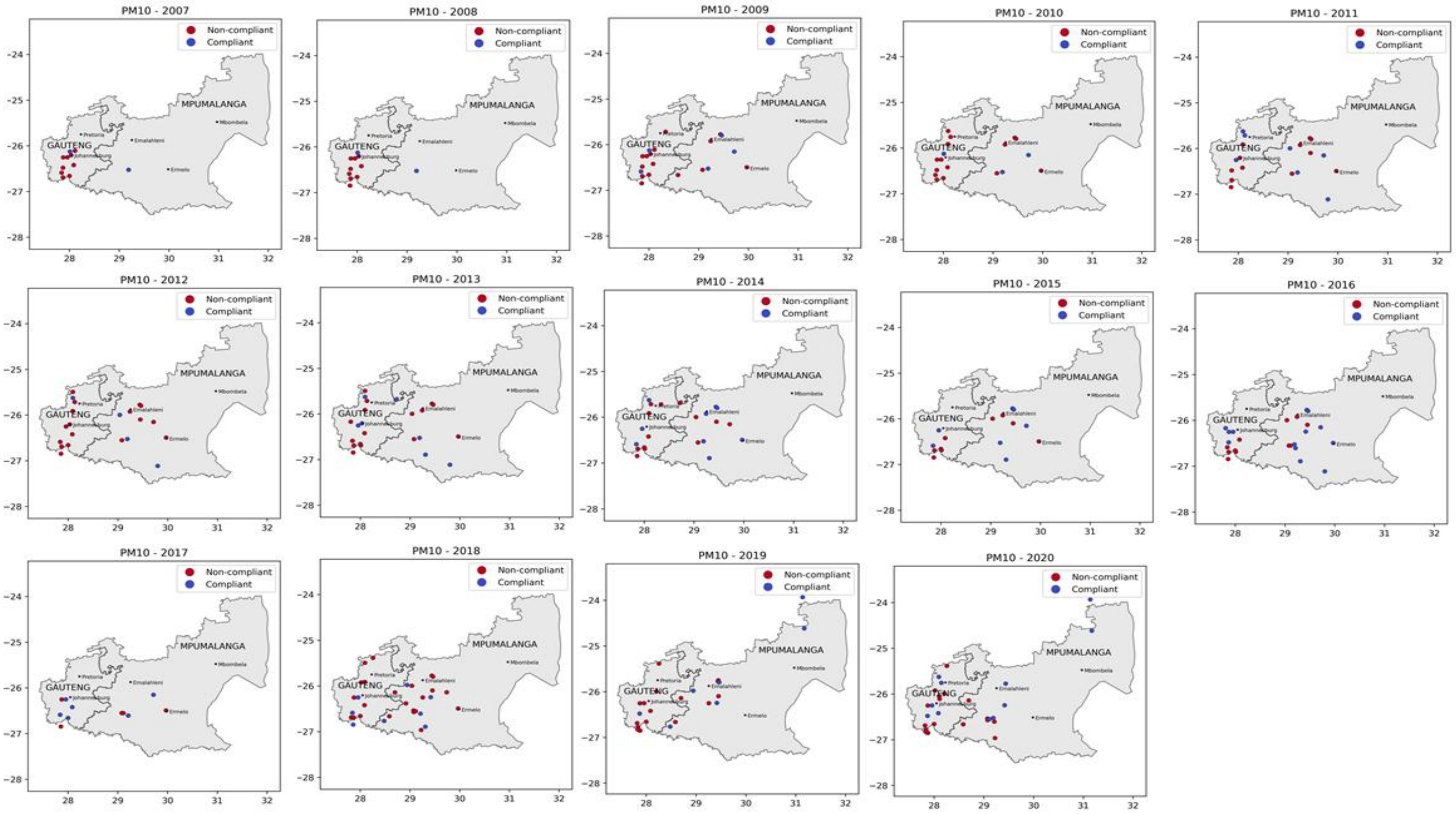

Figure S1. Overview of exceedances (non-compliances) with the PM_{10} NAAQS as monitored at the ambient air quality monitoring stations of the South African Air Quality Information System between 2007 and 2020 in Gauteng and Mpumalanga. The current annual NAAQS for PM_{10} was used to enable direct comparison between years.

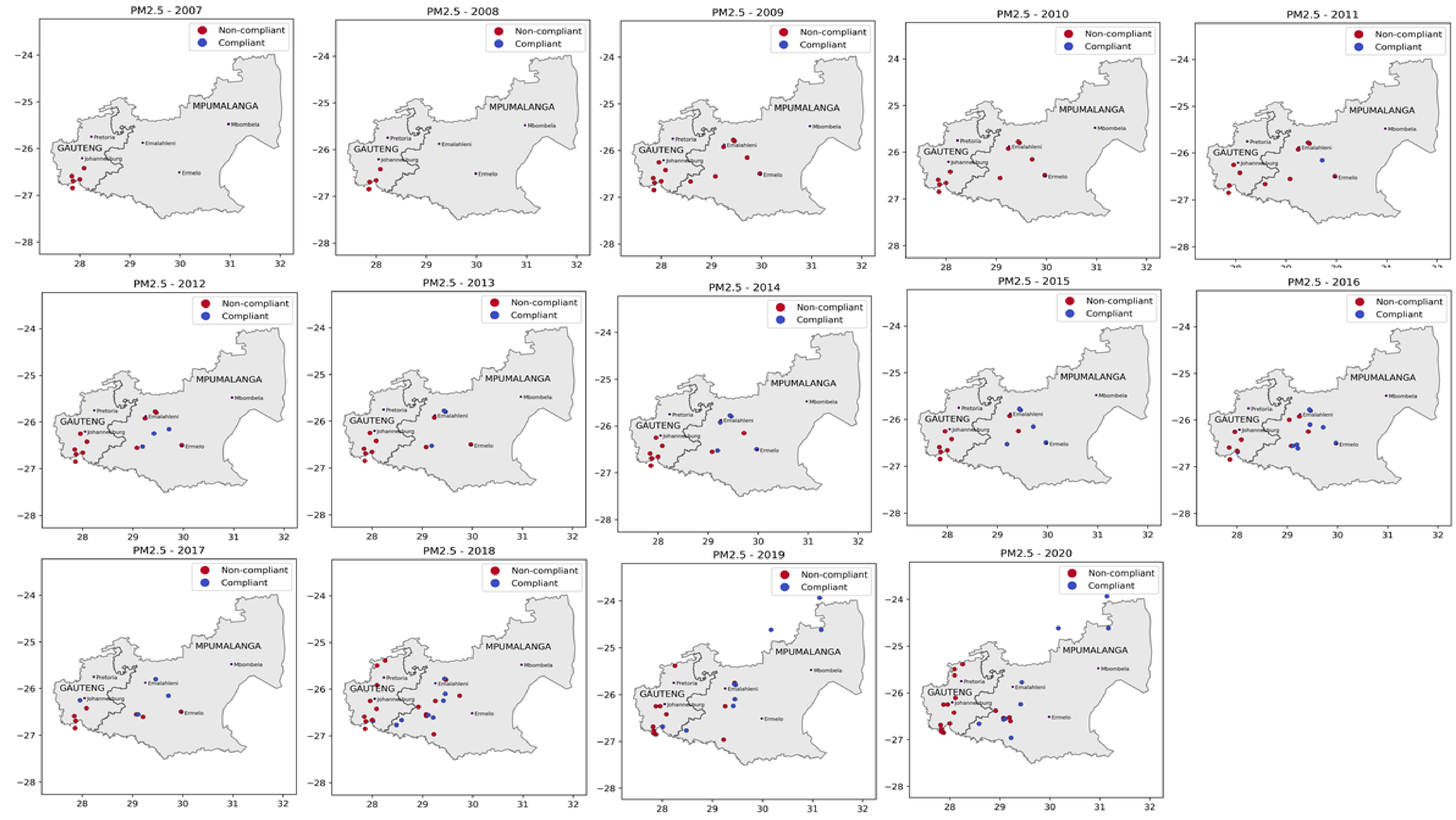

Figure S2. Overview of exceedances (non-compliances) with the $PM_{2.5}$ NAAQS as monitored at the ambient air quality monitoring stations of the South African Air Quality Information System between 2007 and 2020 in Gauteng and Mpumalanga. The current annual NAAQS for PM_{10} was used to enable direct comparison between years.

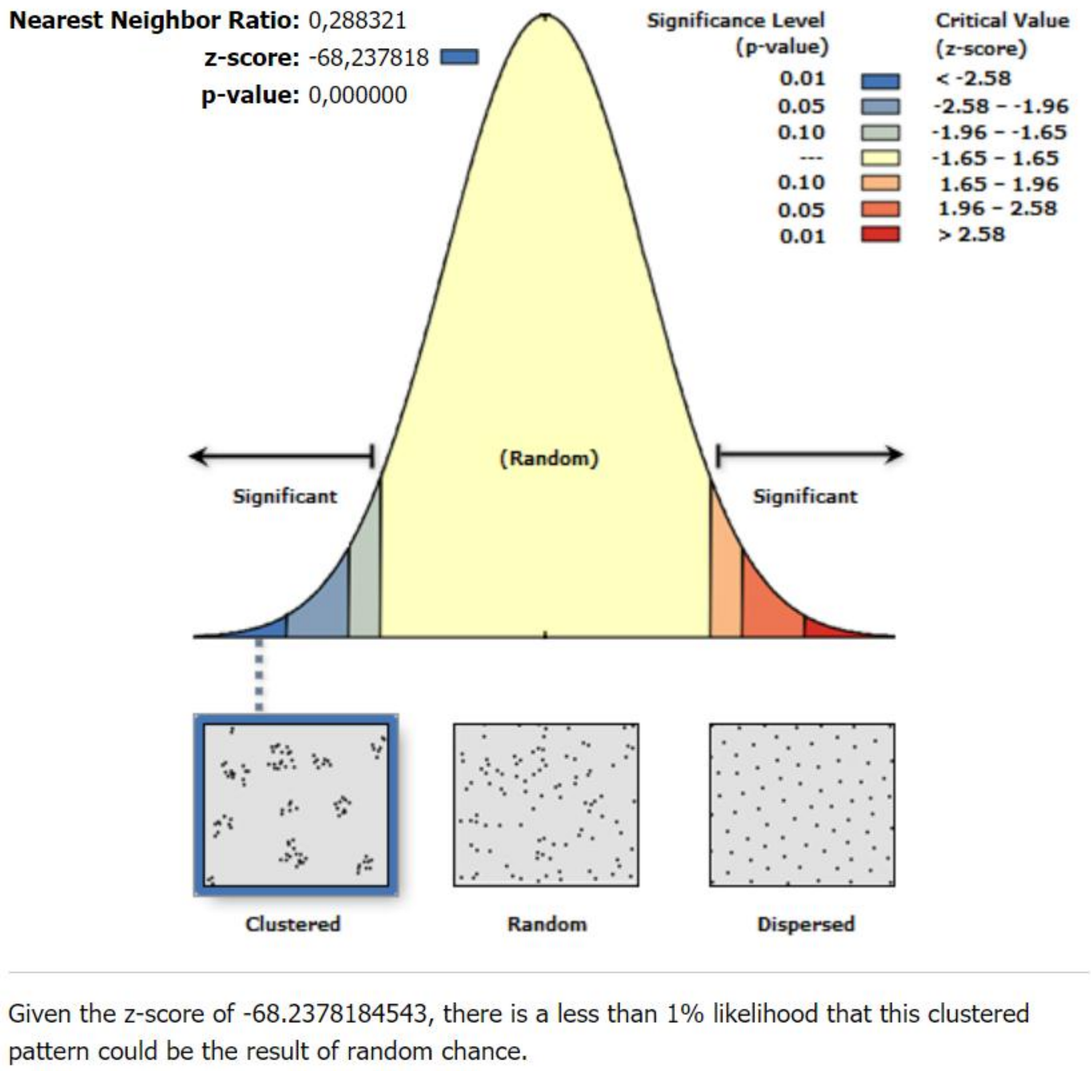

Figure S3: Average Nearest Neighbor statistics; ratio = 0.29, z = -68.24, p < 0.001
